# Novel Low Power Cross-Coupled FET-Based Sense Amplifier Design for High-Speed SRAM Circuits

**DOI:** 10.3390/mi14030581

**Published:** 2023-02-28

**Authors:** G. Lakshmi Priya, Puneet Saran, Shikhar Kumar Padhy, Prateek Agarwal, A. Andrew Roobert, L. Jerart Julus

**Affiliations:** 1 Centre for Innovation and Product Development, Vellore Institute of Technology, Chennai 600127, India; 2 School of Electronics Engineering, Vellore Institute of Technology, Chennai 600127, India; 3 Department of ECE, Francis Xavier Engineering College, Tirunelveli 627003, India; 4 Department of IT, National Engineering College, Kovilpatti 628503, India

**Keywords:** sense amplifier, low power, SRAM, high speed, Modified Cross-Coupled

## Abstract

We live in a technologically advanced society where we all use semiconductor chips in the majority of our gadgets, and the basic criterion concerning data storage and memory is a small footprint and low power consumption. SRAM is a very important part of this and can be used to meet all the above criteria. In this study, LTSpice software is used to come up with a high-performance sense amplifier circuit for low-power SRAM applications. Throughout this research, various power reduction approaches were explored, and the optimal solution has been implemented in our own modified SRAM design. In this article, the effect of power consumption and the reaction time of the suggested sense amplifier were also examined by adjusting the width-to-length (W/L) ratio of the transistor, the power supply, and the nanoscale technology. The exact amount of power used and the number of transistors required by different approaches to better comprehend the ideal technique are also provided. Our proposed design of a low-power sense amplifier has shown promising results, and we employ three variations of VLSI power reduction techniques to improve efficiency. Low-power SRAMs embrace the future of memory-centric neuromorphic computing applications.

## 1. Introduction

High-speed memories, such as Static Random Access Memory (SRAM), are necessary for the massive amounts of information used by today’s digital systems. In an SRAM, a sense amplifier is utilized to identify which data bit (1 or 0) is stored by magnifying the differential input signals to interpret them appropriately. As only a single row of content is accessible per read cycle, each SRAM column needs only one sense amplifier. Integrating differential logic networks reduces their complexity and average delay [1,2,3]. Modeling high-speed sense amplifiers is difficult since each generation of complementary metal-oxide semiconductor SRAMs reduces memory cell size by one-third and increases chip size by 1.5 times, increasing power consumption. Minimal power consumption and strong Complementary Metal Oxide Semiconductor (CMOS) analog VLSI circuits are essential. Scaling down the chip area helps integrate more circuit components in a single chip, thereby reducing the parasitic capacitances, power consumption, and cost of production and increasing the operating speed. Sensing and amplifying data signals are two important functions of a sense amplifier. However, the accuracy of sensing the data signals decreases with a decrease in the supply voltage [4,5,6]. As a result, a large number of memory cells are always busy loading a large number of bitlines, and consequently, the sensing delay increases drastically. Thus this paper initially analyzes the popular and conventional designs and their limitations and proposes a better high-performance sense amplifier design capable of achieving small sensing delay with low power operation. This is achieved by studying and implementing various power reduction techniques to those conventional designs several times to get the final desired model. 

The Differential-Input Body Biased Sense Amplifier (DIBBSA) is designed with au-to-offset mitigation for low-voltage SRAMs, and differential bitline signals are fed to the source and body of the critical sensing transistors [1]. In 65-nm CMOS technology with various operational modes, the usefulness of body biasing in lowering offset is examined. The DIBBSA with floating output nodes (DIBBSA-FL) / DIBBSA with predischarge output nodes (DIBBSA-PD) works consistently at 0.4 VDD from 0 to 75 degrees C. In low-voltage planner CMOS SRAMs, DIBBSA-FL/PD can replace standard SAs [7,8,9,10]. For our proposed design, the negative wordline technique was used to improve power consumption. 

The contribution of the proposed work are listed as follows:

- High-performance sense amplifier circuit for low-power SRAM applications;
- The effect of power consumption and response time of the proposed sense amplifier are investigated by varying the following parameters—Width to Length (W/L) ratio of the transistor, power supply, and different nanometer technologies;
- A comparative analysis of the proposed sense amplifier with different types of existing sense amplifiers, such as differential voltage sense amplifiers, cross-coupled sense amplifiers, latch-type sense amplifiers, etc., has been carried out based on performance metrics.

The remaining sections of the paper are described as follows. Section 2 contains the methodology, design approach, standards used, and constraints of the methodology used in this work. Section 3 discusses the different power reduction techniques used to optimize the existing model, simulation, and the corresponding waveforms on LTSpice software. Section 4 compiles the results obtained after the current work was implemented, and a detailed comparison between all the sense amplifier designs is detailed. Section 5 concludes the paper with discussions about the results obtained and their future implications. 

## 2. Design and Implementation of Sense Amplifier Schemes

This section describes the methodology, design, standards, calibrations, sampling rate, and constraints of the Proposed Sense Amplifier design with an understanding of the base design. 

### 2.1. Basics of SRAM Cell

**Figure 1:**
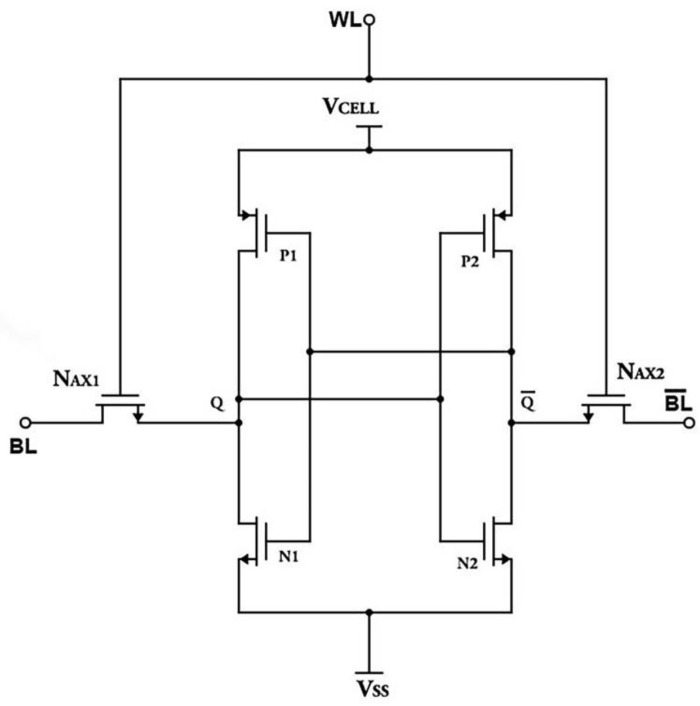
MOSFET-based Static Random Access Memory Cell.

SRAM cells are volatile memory with non-destructible read cycles. Figure 1 depicts a conventional 6T SRAM cell. SRAM cells have two access transistors (N_AX1_, N_AX2)_, two cross-coupled inverters with PMOS (P1, P2) and NMOS drive transistor (N1, N2). Node Q bar stores its complement. The V cell’s (V_CELL_) voltage is used to power the entire circuit. The SRAM cell will maintain its value eternally as long as the voltage is delivered to the circuit through the V cell. The wordline (WL) enables any read-to-write action in the circuit. Unselected wordline puts the SRAM cell in standby mode. When you select an address, the wordline status becomes high, selecting all its cells.

This allows the access transistors to unlock the cells from standby state, allowing them to read and write. The cell depicted above also has two bitlines that govern the cell’s data input and output. The first line, the bitline (BL), contains the same value as the cell, and the second bitline bar (BL bar) contains the inverse value.

- Read and Write Peripherals of Sense Amplifier

As we can solely access one row at a time and another cell per column, read and write peripherals are highly required. All SRAM cells in a column can share the same peripheral, maximizing efficiency.

- Write Circuitry

When writing a value into the cell, bitlines are the method of choice. At node Q, the cell’s value is saved, while at the node Q bar, the value’s inverse is retained. According to the evidence demonstrated by the circuit, logic 1 and logic 0 are not written at the nodes in their respective forms; rather, just logic 0 is written at the node Q or Q bar, depending on the value that is required. To write the value into the cell, one of the bitlines should be held at the value of Vdd, while another bitline must still be held at the value of Vss. Doing this will cause the cell to function like a latch.

- Precharge Circuitry

**Figure 2:**
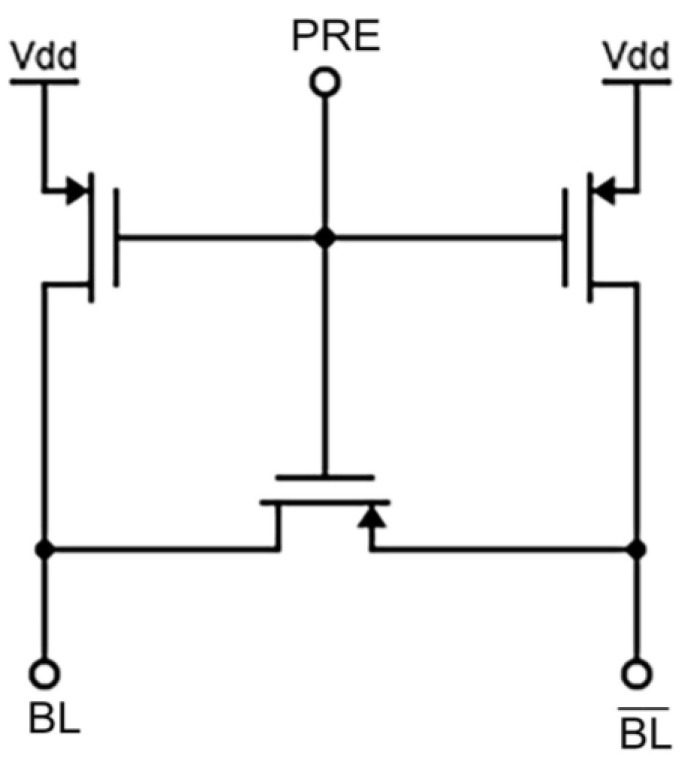
Sample Precharge circuit.

Before starting the reading cycle, this circuit balances the voltage on both bitlines. Three transistors, either NMOS or PMOS, make up the basic circuitry of a precharge. Two of the transistors connect the bitlines to Vdd, while the third connects the two bitlines to maintain a constant voltage across them both. Three PMOS transistors are shown in Figure 2 as part of a precharge circuit. In this instance, the bitlines are precharged by dropping signal PRE to zero, which causes the PMOS transistor to switch ON and the bitline voltage to surge to Vdd.

### 2.2. Basics of Sense Amplifier

**Figure 3:**
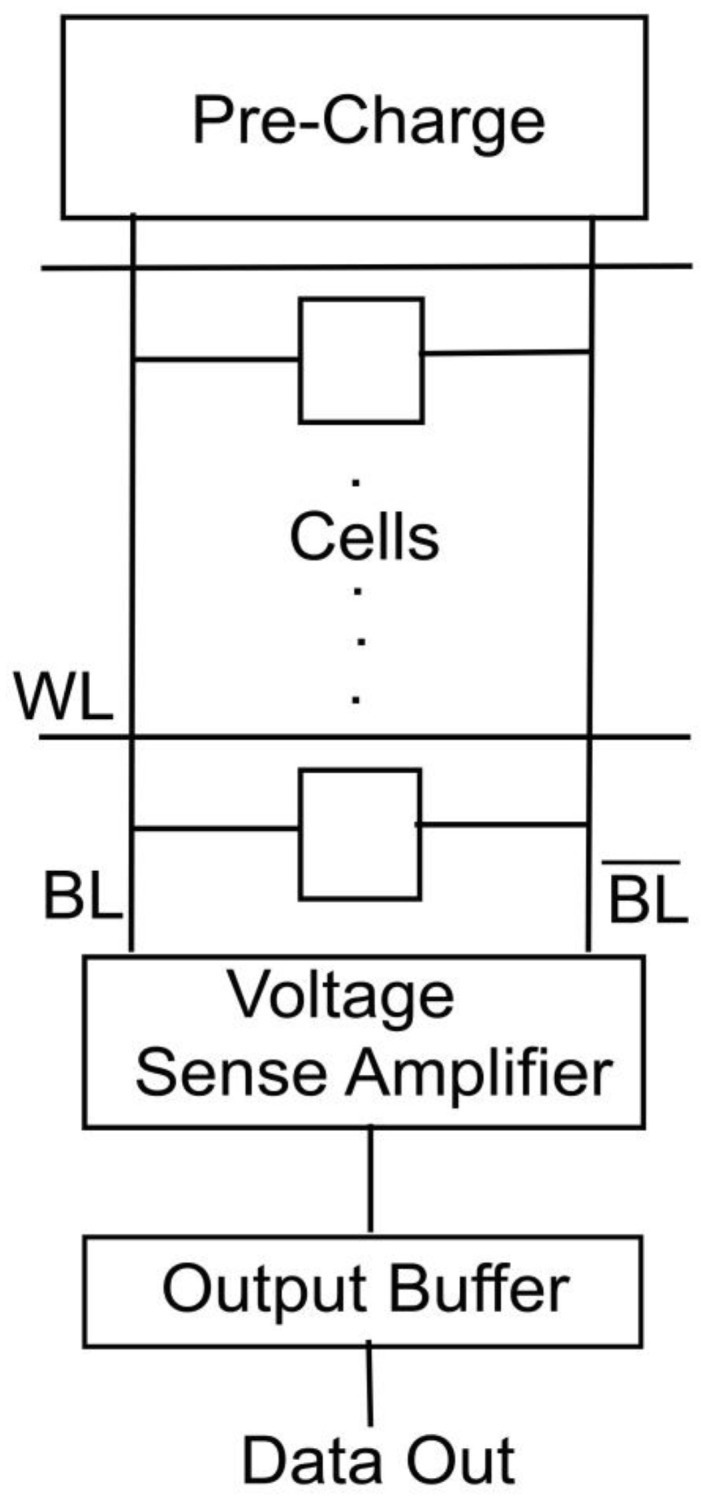
SRAM column with Sense Amplifier.

Sense Amplifiers are fundamental to modern computers. It’s a basic IC memory chip component. The word derives from magnetic memory [3]. A sensing amplifier reads information from memory. The main role is to read a low-power signal produced from the bitline indicating data bits (1 or 0) held in the memory section and magnify the signal or tiny voltage swing to identifiable logic levels so the data may be captured and used [3]. Today’s sense amplifier designs use 4–6 transistors, especially in contrast to the 10–14 used before. In general, hundreds or thousands of almost the same sense amplifiers are deployed for a single application. Memory cells store information on the chip. Generally employed in volatile memory cell sections. Figure 3 shows rows and columns of SRAM and DRAM memory cells. 

A line has been fixed to each cell in the memory called wordlines that are activated using a particular voltage level in those lines. The voltage level for each wordline is set by the technology node selected for the transistor design. This article mainly considers the sense amplifier implementation at 180 nm, for which the voltage value set will be at 1.8 V. Each sense amplifier is attached to two such lines, which are complementary to each other. Each cell automatically lies so that there is an interaction between the wordline and the bitline. Data are written and read by the same bitlines. SRAM and DRAM operations are the two types of operations performed by sense amplifiers:

- Operation of the SRAM:

The wordline has been set to high in reading mode, which activates the cell in that row. 0 and 1 are the saved values. The differential signal is amplified to normal logic level by a sense amplifier placed at the end of the lines. The requested cell’s bit is then latched into the buffer and sent to the output bus.

- DRAM operation:

It performs a similar function to SRAM, except that information in the DRAM chip is held as electric charges in small capacitors within a memory cell. Because reading data from a cell depletes charge and causes the data to be disrupted, the data are instantly written back into the cell by applying voltage and recharging the capacitor. Memory refresh is the term for this process.

The basic goal of the sense amplifier is to reduce sensing delay, increase amplification, reduce power consumption, fit in the allowed space, and have a high level of dependability.

### 2.3. Types of Sense Amplifiers

The overall implementation for all the designs is done in Linear Technology Spice (LTSpice) software. All conventional designs, such as the differential voltage sense amplifier, basic latch sense amplifier, and basic latch sense amplifier with pass transistors, are designed. Their corresponding output waveforms are shown below. 

#### 2.3.1. Differential Sense Amplifier

**Figure 4:**
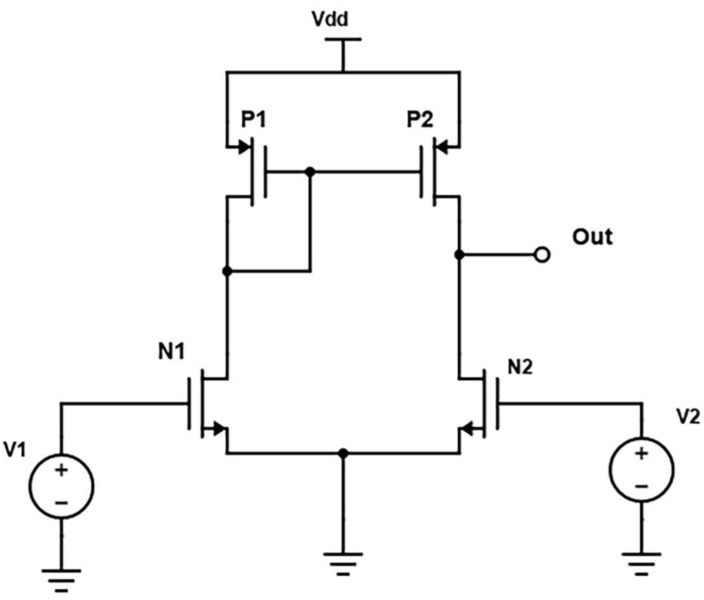
Differential Sense Amplifier Schematic.

**Figure 5:**
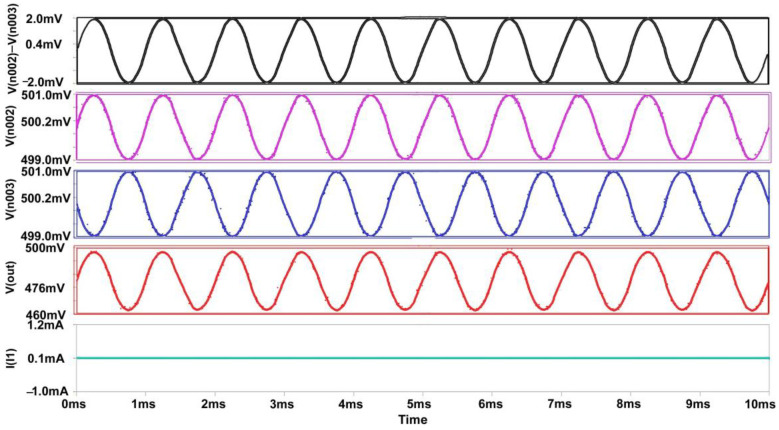
Output waveform of Differential Voltage Sense Amplifier.

The differential amplifier circuit presented in Figure 4 is the most standard design form currently being used. This dynamic voltage mode amplifier consists of a differential pair with active current mirror load (P1, P2–PMOS Transistors) attached at the end of the biasing current source or ammeter [8]. Like all the amplifiers, the differential pair transistors (N1, N2–NMOS Transistors) gates are connected to bitlines, and output is obtained from the similar side of the V2. V1 and V2 are the supply voltages to the NMOS transistors. As the current source is connected at the end, we can expect that at every instant the sum of the current flowing from both transistors will be constant and equal. The transient response of the differential amplifier is plotted in Figure 5. As the gate-to-source voltage of both PMOS transistors is identical, the input current becomes the same as the output current, which is observed to be static to accelerate the sensing action at a faster pace but with the drawback of experiencing 179 µW.

#### 2.3.2. Basic Latched Sense Amplifier

**Figure 6:**
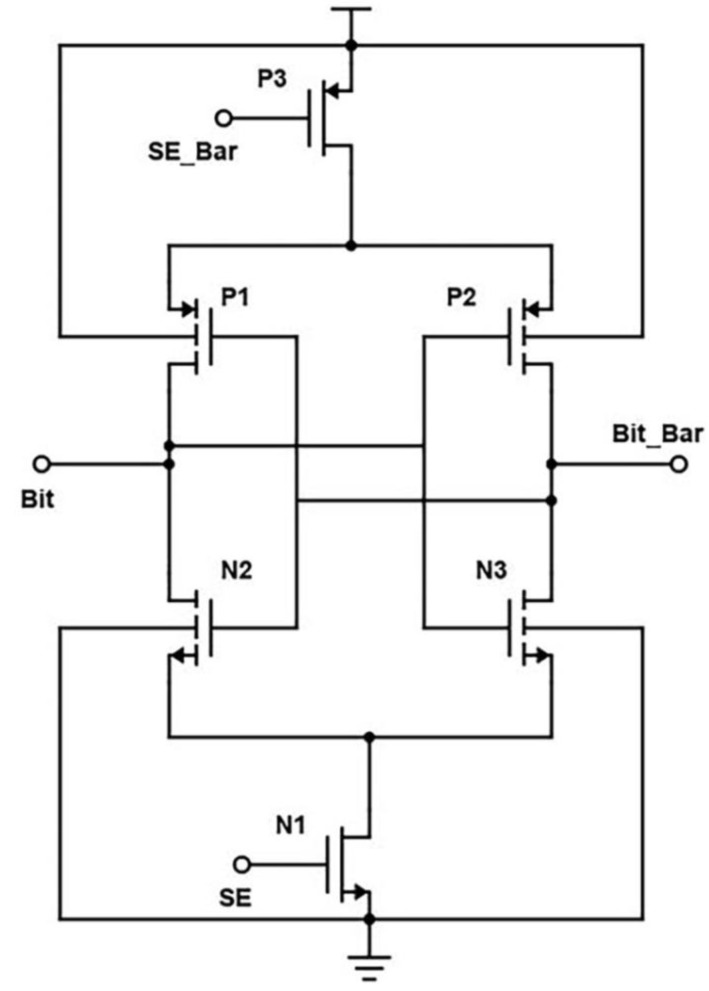
Basic Latched Sense Amplifier Schematic.

**Figure 7:**
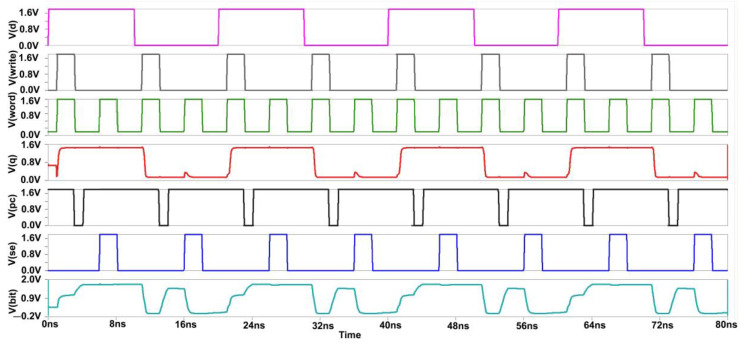
Output waveform of Basic Latch Sense Amplifier.

The differential amplifier is a dynamic type, but the basic latch sense amplifier is the static voltage mode sense amplifier design. Two cross-coupled inverters make positive feedback which enables the latching behavior, as shown in Figure 6. In the read cycle, before it starts, the bitlines are precharged to a certain voltage level to start at a common point so that the voltage difference will later be clearly sensed [7]. These discharging bitlines are connected to the inputs of the sense amplifier, which allows the development of small voltage bias at the gates of the MOSFET before inverters are turned on, and the sense amplifier is enabled. The response of the basic latch sense amplifier is shown in Figure 7.

#### 2.3.3. Basic Latched Sense Amplifier with Pass Transistors

**Figure 8:**
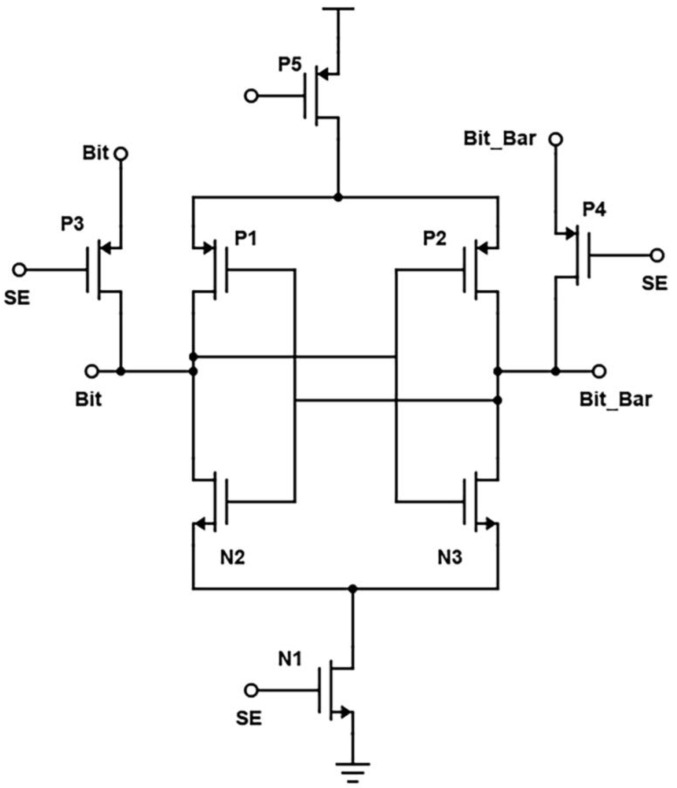
Basic Latched Sense Amplifier with Pass Transistor Schematic.

**Figure 9:**
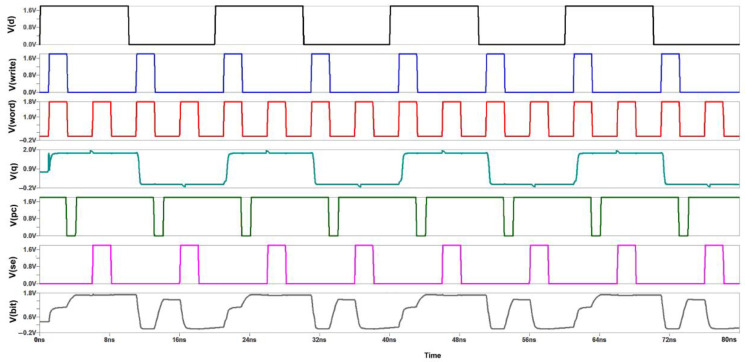
Output waveform of Basic Latch Sense Amplifier with Pass transistors.

The amplifier in Figure 8 has the same fundamental functionality as the preceding sense amplifier, but the addition of the pass transistor separates the inputs and outputs of the amplifier from the bitlines. As a logical cause, the sensing amplifier will endeavor to drain the bitlines anyway before attempting to store the input data in the SRAM. Since the pass transistor is still active whenever the sense amplifier is disabled, the voltage on the gate can continue to develop even though the sense amplifier has been turned off and the bitlines are discharging. As soon as the sense amplifier is powered on, the pass transistor deactivates down, and the bitlines become decoupled from the amplifier. After the sensing amplifier has been enabled, bitlines cannot be discharged any longer. The corresponding transient response is shown in Figure 9. The downside is that the pass transistors are isolated from the other transistors. 

### 2.4. Cross-Coupled Sense Amplifier

**Figure 10:**
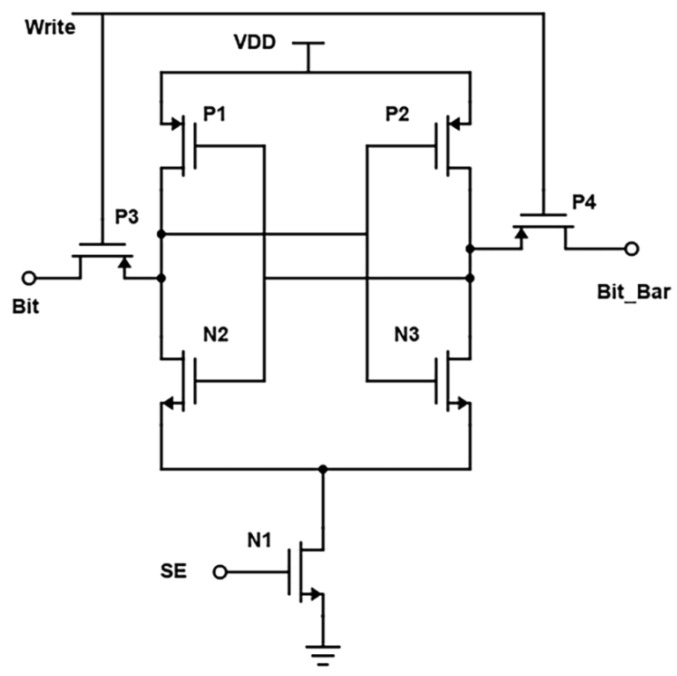
Schematic of the cross-coupled sense amplifier.

Transistors P1, P2, N2, and N3 form a current conveyor as a column sector, as in Figure 10. When the Bit signal is low while the cell is accessed for turning ON the transistors M2 and N3, the differential signal will flow from the bitlines to the data lines [1]. From the perspective of a circuit that operates in voltage mode, P1, P2, and N2, N3 build up two MOS amplifiers. The yields of such amplifiers are proportionate to the equivalent resistance of N2 and N3. The two amplifiers in the current conveyor circuit are interconnected to one another using positive feedback. At the termination of the reading operation, the column select signal CS transitions from a low to a high state. This causes an increase in the equivalent resistance of N4 and N3, as well as in the gains of the MOS amplifiers. After the reading procedure, the high-efficiency amplifiers will amplify the voltage difference between nodes rather than eliminate it automatically. 

**Figure 11:**
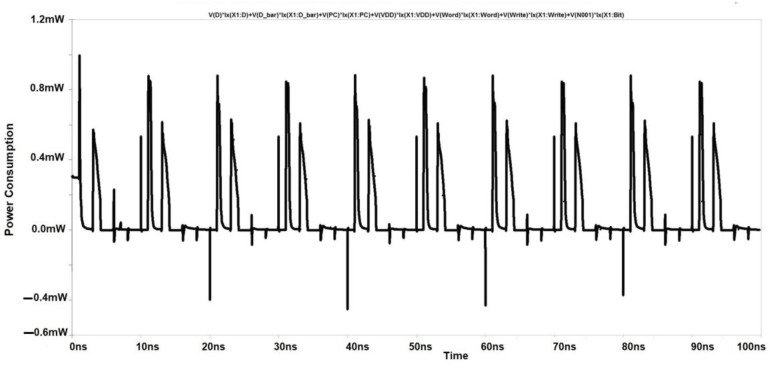
Power Consumption Spectrum of Cross Coupled Sense Amplifier.

Simulations with varying sizes of transistors demonstrate that the cross-coupled transistors P1 and P2 largely lock this unbalanced configuration, even when the column select signal CS drops again. In the previous study, it was believed that nodes had equivalent voltages. [1]. This 6T-SRAM circuit is designed using CMOS inverters. This design helps them in making the least power dissipation. At the micro-scale, the major challenge is the leakage current. Output terminals are used as internal lines or bitlines to store bits of data. The output terminals complement each other in this design, as shown in Figure 10. The power consumption spectrum of the cross-coupled sense amplifier is shown in Figure 11.

During the write operation, data are provided at these connections, as the data bit is written using the bitline orbit, and an inverted logic value is provided on bit_bar. The wordline is turned on. Then the bitlines overpower the cell with new values if out = 0, out_bar = 1, and bit = 1, bit-bar = 0, which forces out to high and out_bar to low. During the read operation, logic values are addressed to bit and bit_bar.

Wordline is kept high to enable sharing of charge between lines. If out = 0 and out_bar =1, the bitline discharges through the two adjacent NMOS transistors and ground, and the bit_bar line stays high. The cross-coupled regeneration response and the presence of other devices in the circuit pose problems for this design. For example, the presence of an equilibrium device between two differential nodes and a tail current source can drastically reduce the circuit’s performance. 

## 3. Implications of Power Reduction Strategies in Standard Sense Amplifier Design 

### 3.1. Power Reduction Techniques

The next sections explore some circuit-level leakage power reduction approaches for a 6T-SRAM cell. All are accomplished by manipulating the SRAM cell’s terminal voltages in standby mode [11,12,13,14,15,16].

#### 3.1.1. Sleep Transistor Technique

PMOS transistor is connected between VDD and SRAM cell, and NMOS transistor is connected between SRAM cell and GND. Both sleep transistors are turned on in active mode, creating a route between VDD and GND. Because both of these transistors are switched off in sleep mode, the power supply to the SRAM cell is shut down, resulting in a virtual VDD and GND path.

#### 3.1.2. Source Biasing Scheme

In this method, a pull-down NMOS transistor with its gate terminal attached to the wordline is connected between GND and the SRAM cell. The wordline goes high in active mode, and the NMOS transistor is turned on. Because of its low resistance, the virtual ground voltage VSL acts almost identically to a real ground line, and the SRAM cell performs normally. The wordline is set low in standby mode, which shuts off the NMOS transistor and raises the source voltage while lowering the subthreshold and gate leakage current.

#### 3.1.3. Negative Wordline Scheme

During idle time, a negative voltage is applied to the wordline without compromising the device’s performance. The threshold voltage is raised while the leakage current is lowered whenever a majority of NMOS are driven with a negative bias or the majority of PMOS are forced with a positive bias. It has been discovered that keeping the bias below −0.4 V is adequate to reduce the leakage current [11].

#### 3.1.4. Dual Sleep Technique

In this method, two pairs of transistors each (PMOS, NMOS) are connected in parallel. The initial pair is between VDD and the SRAM cell, while the second is with the SRAM cell and GND. The sleep transistors (NMOS and PMOS transistors) are turned on in active mode, creating a route between VDD and GND in the same way that the sleep transistor approach does. The NMOS and PMOS transistors are set ’OFF’ in "sleep mode," which shuts down the power supply to the "SRAM" cell and creates a virtual VDD and GND path [2].

### 3.2. Modified Standard Sense Amplifier Implementations: Average Power Consumption

**Figure 12:**
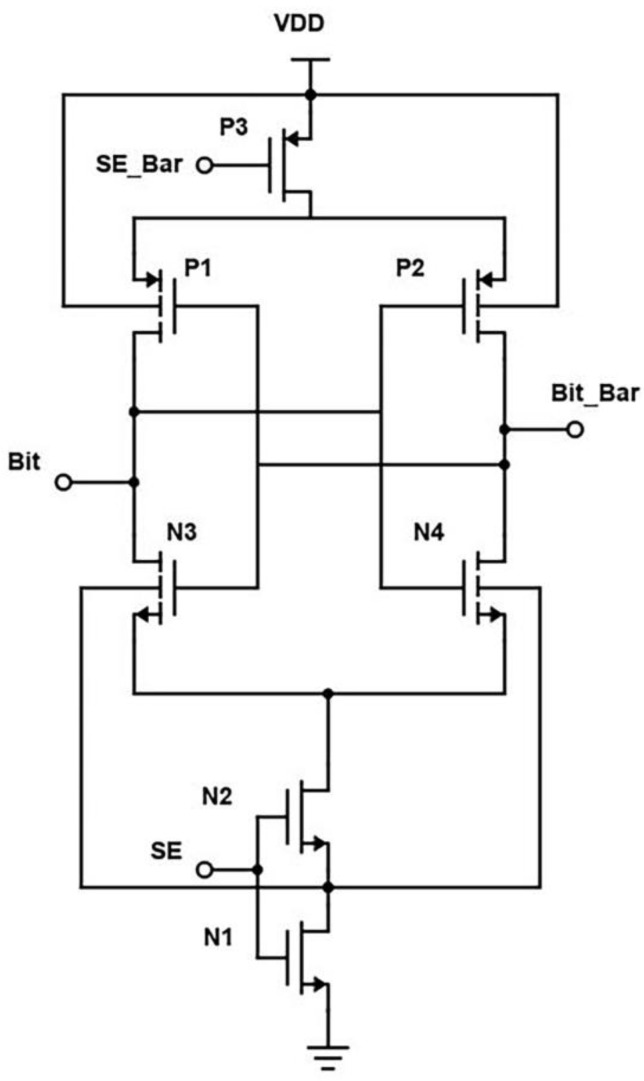
Modified Basic Latch Sense Amplifier Schematic.

**Figure 13:**
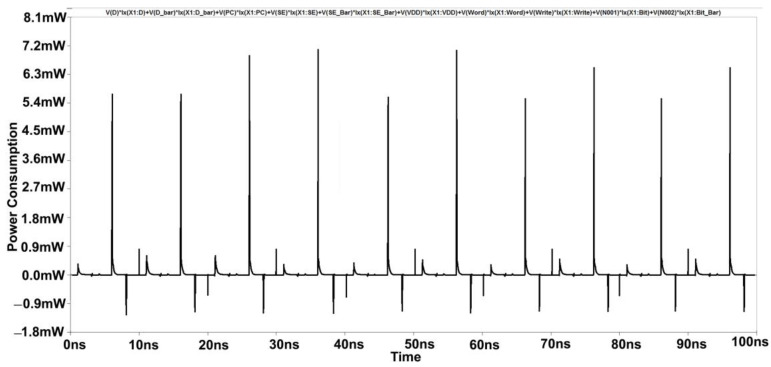
Power Consumption Spectrum of Modified Basic Latch Sense Amplifier.

The sense amplifier designs were analyzed using 180 nm MOS technology. Following Table 1 show the average power consumed with varying supply voltage. We can observe that as we increase VDD (supply voltage), the power consumed by all the sense amplifier designs also increases. The basic latch sense amplifier design schematic is shown in Figure 12, with its corresponding power consumption spectrum depicted in Figure 13. To maximize throughput and minimize power consumption, the sense amplifier here provides a fast differential signal reading on the bitlines. The power consumption still lies close to 7.2 mW. 

**Figure 14:**
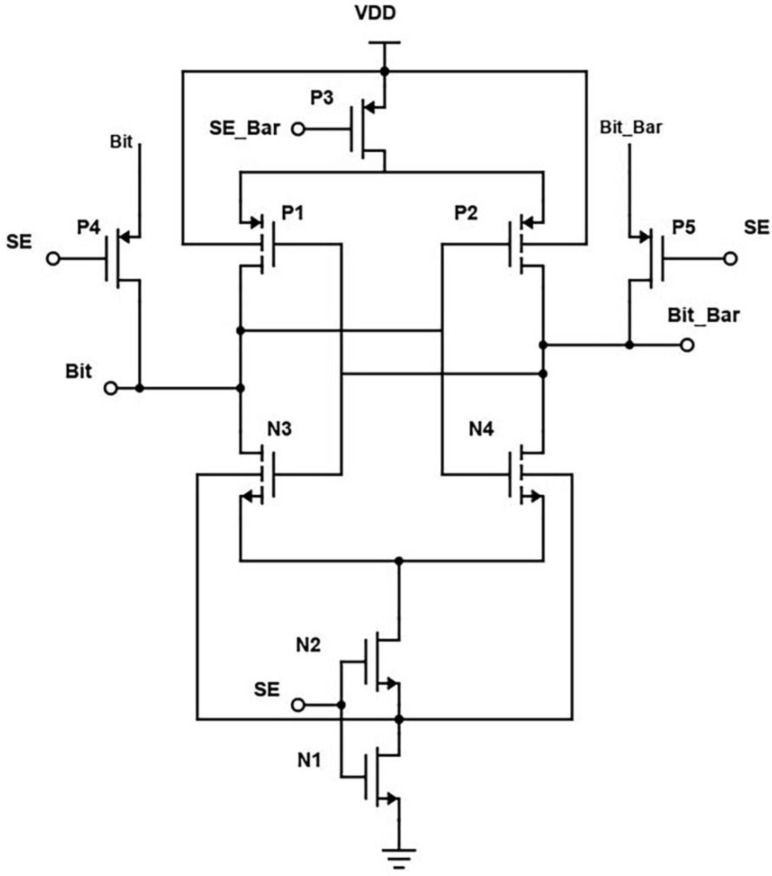
Modified Basic Latch Sense Amplifier with Pass Transistor Schematic.

**Figure 15:**
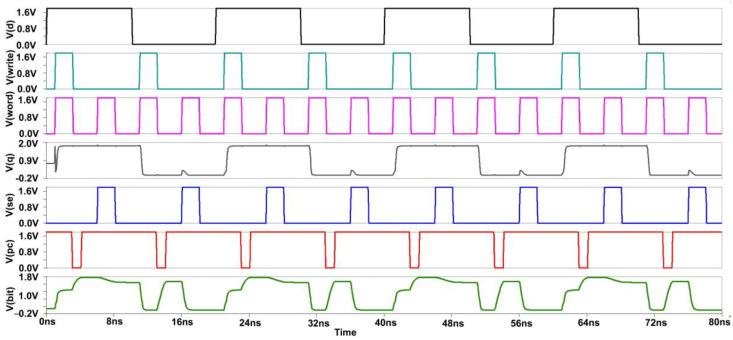
Output Waveform of Modified Basic Latch Sense Amplifier with Pass Transistor.

However, with the inclusion of pass transistors in the basic latch sense amplifier design, as shown in Figure 14, the amplifier inputs and outputs are decoupled from the bitlines, which removes the probability of the sense amplifier attempting to read the SRAM value incorrectly retained in the cell after the bitlines are completely discharged due to the same physical nodes in the former design. Although the basic latch sense amplifier design with the pass transistors has the advantage of the presence of dissimilar physical nodes, including the two NMOS transistors on either side adds the voltage drop across them, as plotted in Figure 15. Consequently, this may result in biased logic values because of imprecise fabrication of the transistor size. 

**Figure 16:**
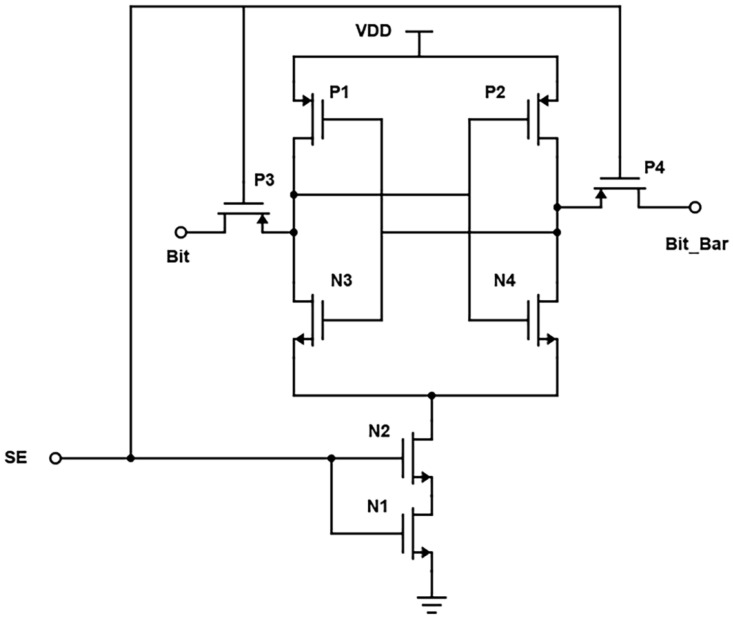
Commonly Cross Coupled Sense Amplifier Schematic.

**Figure 17:**
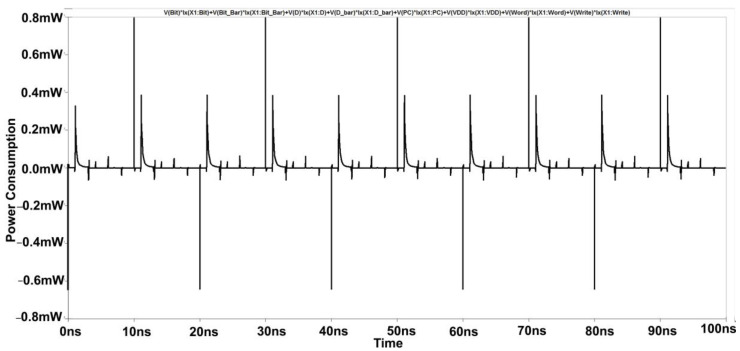
Power Consumption Spectrum of Commonly Cross Coupled Sense Amplifier.

Figure 16 shows the commonly cross-coupled sense amplifier design, highlighting the common connection of the "Sensing Enable (SE)" signal. This design schematic reduces the transistor count through which separate write signal and sensing enable signals will be provided, as shown in Figure 10. Figure 17 depicts the corresponding reduction in power consumption to 0.8 mW at 180 nm. 

Table 1 compares average power consumption with the corresponding transistor sizing of all three conventional designs. Moreover, the power reduction techniques used for our proposed design, i.e., source biasing and negative wordline, were also implemented on the conventional designs, and the power readings and their transistor sizing were noted and tabulated in Table 2.

## 4. Proposed Sense Amplifier: Results and Discussion

We can categorically state that the fundamental function of sense amplifiers is to magnify the small differential voltage difference between the bitlines into a logically high value based on the preceding magnificent overview of sense amplifiers. The bitlines have a high value of the capacitive load, which in turn offers RC delays. The recent SRAM designs [17,18,19,20,21,22,23,24,25,26] reported depict the novel device architectures of 6T SRAM with FinFET, Memristor, and Junctionless TFETs, to claim it to be a low-power device. Here, with our proposed sense amplifier design, the power reduction techniques like negative wordline and source biasing were combined and utilized.

Also, from the previous designs, we discussed that sense amplifiers include a differential logic circuit to enhance the complexity of logic circuits and minimize the delay faced by the differential circuit [13]. The main challenge is during the designing of the large-scale SRAMs in CMOS technology because large SRAMs will come with N number of bitlines, resulting in a large delay [11].

Another challenge comes when the designs used to be operated under a value that is lower than the supply voltage because the main objective is fast sensing, and this is not being fulfilled [4]. The most widely used design is the cross-coupled sense amplifier. The complementary structure of the cross-coupled inverter is made from two pull-up and two pull-down transistors. The above-mentioned design’s speed and loading characteristics are based on discharging conductivity and cross-coupled capacitance values. The conductivity is inversely proportional to the capacity; that is, it produces large conductivity for minimum capacity.

**Figure 18:**
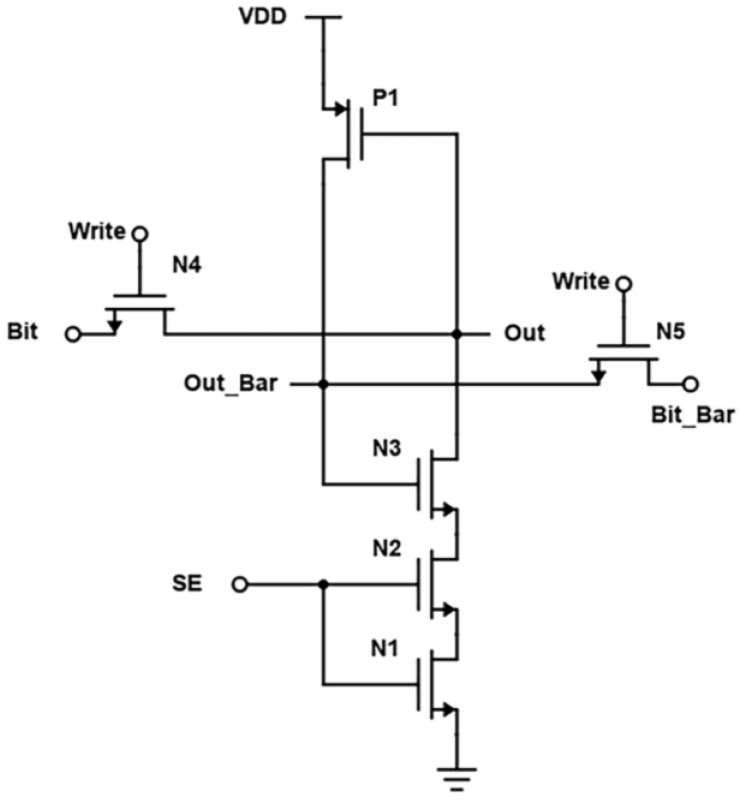
Schematic of Proposed design (Modified Cross-Coupled SRAM).

In the proposed design, the two cross-coupled inverters are replaced by two transistors, as in Figure 18. When the wordline is activated, the transistors N4 and N5 are turned on, and the bitline voltage is passed to the output terminals. To further reduce the power consumption, a technique to reduce leakage current called source biasing basically means attaching an NMOS at the end of the circuit operated using sense enable, which provides a virtual ground while switched on. Source biasing is a power reduction, or, we can say, a leakage current reduction technique, which generally means that between the ground and source lines of the SRAM cell, a pull-down NMOS transistor is introduced, and its gate terminal is connected to the wordline [4]. The transistor is turned on after the WL becomes high in active mode. Because of its low resistance, the virtual ground voltage VSL practically acts as a true ground line, allowing the SRAM cell to function normally. The source voltage is raised and the subthreshold and gate leakage currents are reduced in standby mode because WL is set low and N4 is turned off.

**Figure 19:**
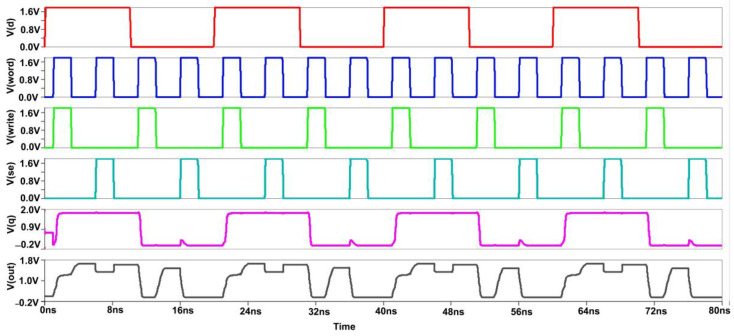
Output Waveform of Proposed Sense Amplifier.

We see that low-power measures are also lower access time measures for large memories. The cross-coupled FET configuration, along with negative wordline and source biasing techniques, the bitlines of the proposed design have less capacitance effect, thereby offering reduced RC delay. This is evident from the reported "Modified Cross-coupled design", as shown in Figure 19. Back to the proposed design, while in READ mode, we also incorporated the negative bitline technique, which is used for the same purpose as source biasing, i.e., generating a negative voltage supplied to the wordline during the idle time without affecting the device performance or Soft Error Rate (SER) [4]. As the access transistors are turned off, the sub-threshold leakage current is reduced drastically in turn. But one disadvantage of this technique is that it promotes the gate leakage current of the access transistor due to enlarged gate-source and gate-drain voltage differences. Basically, this technology stabilizes the write signal value between −0.9 to 0.9. As a result, after incorporating these two designs in the modified, the power is greatly reduced. 

**Figure 20:**
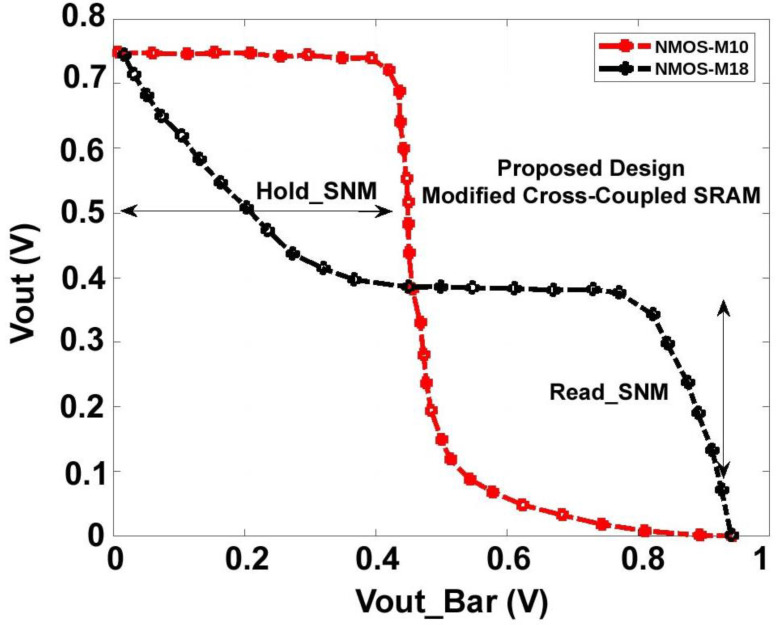
Hold and Read Static Noise Margin (SNM) of Proposed Sense Amplifier.

**Figure 21:**
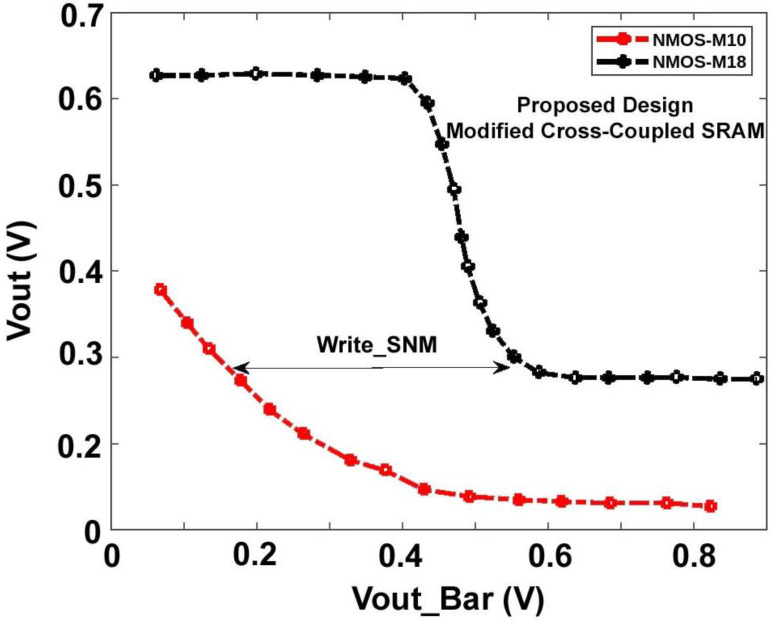
Write Static Noise Margin (SNM) of Proposed Sense Amplifier.

**Figure 22:**
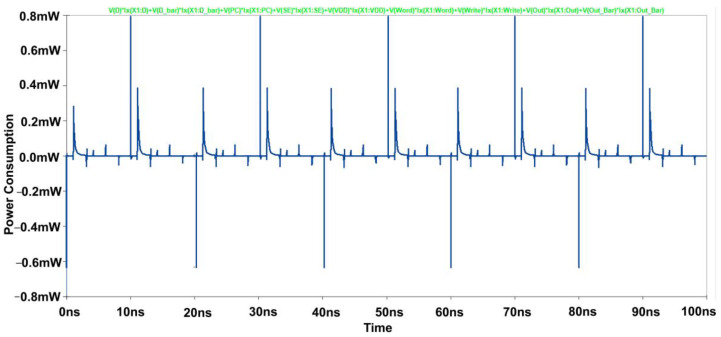
Power Consumption Spectrum of Proposed Sense Amplifier.

Figure 20 and Figure 21, shows the hold, read, and write Static Noise Margin (SNM) measurements for the proposed sense amplifier configuration of static RAM. The results indicate that the recommended device has good noise endurance because read and write operations require correct SNM window frames. Also, a higher noise margin indicates the worth of signal quality. Compared to the circuit’s propagation delay time, a pulse width of a few microseconds is unusually long for high-speed ICs. Hence, a reasonable noise margin in the proposed design provides lesser delay and makes the Modified Cross-Coupled sense amplifier more convenient for implementing high-speed SRAM circuits. The average power consumption spectrums for both waveforms are also shown below in Figure 22.

Initially, for the write cycle, when the word and write signal are at HIGH input (logic 1) simultaneously, then the data are copied from data D to Q. For the read cycle, we want to precharge the circuit first and enable the SE (sensing enable). On the left-hand side of the SRAM cell, when we are writing logic 1, the bitline is not fully charged to VDD (1.8 V) because the NMOS transistor does not transmit perfect 1 or strong 1. Thus, two voltage drops will occur due to the threshold voltage of the two transistors at the Q output, while on the other side, the bitline will be charged to full 0. So the difference will be sufficient to toggle the cell into the required state. The PC (precharge) signal is given logic 0, so the PMOS transistor can transmit full 1 or strong 1, and the bitlines will be charged to full VDD. For the read operation, the wordline and SE signal both should be at HIGH logic simultaneously to copy the data stored at the Q output to the bit output [9].

In Table 3, the modified column shows the power reduction techniques implemented in the conventional designs. The inclusion of the source-biased transistor and the power is reduced for all the cases. The modified cross-coupled sense amplifier has an average power consumption of 7.5912 μW close to our proposed design’s power, which is 7.4412 μW, but the transistor sizing area is less in our proposed design, which makes it the best optimal high-performance and low-power sense amplifier suitable for high-speed SRAM applications.

**Figure 23:**
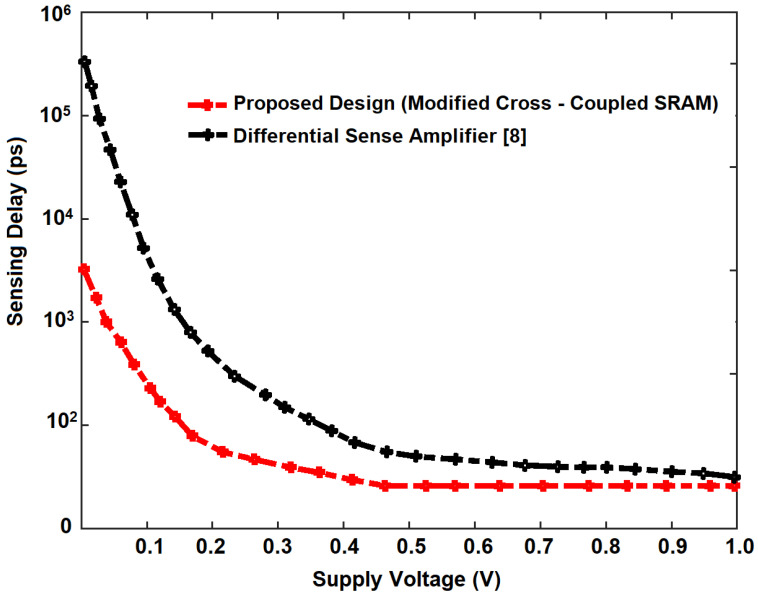
Sensing Delay of Proposed Sense Amplifier.

The performance of the sense amplifier depends mainly on the overall sensing delay, depicted in Figure 23. As the proposed design of Modified Cross-Coupled SRAM has imbibed the negative bitline and source biasing techniques, the sensing delay is less when compared to the differential sense amplifier [8]. Initially, when the supply voltage is 0.1 V, the proposed design shows a prominent (almost 99%) downshift in the sensing delay due to the reduced capacitance effect on the bitlines, significantly lowering the latency. The reduced capacitance effect is due to applying negative voltage to the wordlines without affecting the noise margin, power, and delay. With varying supply voltage from 0.1 V to 1.0 V, the sensing delay has almost reached from 5.5 ns to 55 ps. 

## 5. Conclusions

In this paper, several conventional sense amplifier designs and their average power consumption with varying parameters, such as power supply, were studied. Power reduction techniques like sleep technique, negative wordline, source biasing, etc., were analyzed and implemented on base designs to get our proposed model. Limitations, constraints, trade-offs, and alternatives like input/output voltage levels, propagation delay, static current characteristics, and slew rate were also analyzed. The leakage current of the SRAM cell-access transistors connected externally to the bitlines was kept minimum as long as the wordline voltage was below −0.4 V. Hence, the average power consumption of our sense amplifier design in the standby mode was drastically reduced. The power reduction techniques were implemented on the popular/conventional designs. The proposed sense amplifier was also compared with all the other conventional designs of note, making it an optimal design for high-speed SRAM applications. With the help of our proposed sense amplifier design, digital systems can use this high-memory SRAM design to store and retrieve large amounts of data. Due to the lesser transistor sizing area, lesser bit and data line capacitances will help achieve higher sensing speed in integrated circuit technology.

## Figures and Tables

**Table 1:** Comparison of average power consumption with varying supply voltage for different sense amplifiers.

Sl. No.	V_DD_ (V)	P_DC_ (μW) for 180 nm			
		Differential Voltage Sense Amplifier [8]	Basic Latch Sense Amplifier [7]	Basic Latch Sense Amplifier with Pass Transistors [5]	Commonly Cross Coupled Sense Amplifier [1]
1	1.2	119.92	36.425	36.627	36.481
2	1.5	149.93	55.412	55.656	52.983
3	1.8	179.94	80.713	81.061	77.097
4	2.1	209.95	95.995	92.227	101.18
5	2.4	239.96	107.770	112.40	135.63

**Table 2:** Comparison of average power consumption with supply voltage and their transistor sizing for all three conventional designs.

Sl. No.	Name of the Design	Supply Voltage (V)	No. of Transistors Used	Average Power Consumption (μW)
1.	Differential Sense Amplifier [8]	1.8	4	179.94
2.	Basic Latched Sense Amplifier [7]	1.8	6	80.713
3.	Basic Latched Sense Amplifier with Pass Transistors [5]	1.8	8	81.061

**Table 3:** Comparison of average power consumption for all the designs with their modified versions.

Sl. No.	Name of the Sense Amplifier Design	Conventional		Modified	
		Area (Transistors)	Power Consumed (μW)	Area (Transistors)	Power Consumed (μW)
1.	Basic Latch Sense Amplifier [7]	6	80.713	7	39.504
2.	Basic Latch Sense Amplifier with Pass Transistors [5]	8	81.061	9	9.46
3.	Cross-Coupled Sense Amplifier [1]	7	77.097	8	7.5912
4.	Proposed Design	5	80.64	6	7.4412

## Data Availability

Not applicable.
